# Memory-Augmented 3D Point Cloud Semantic Segmentation Network for Intelligent Mining Shovels

**DOI:** 10.3390/s24134364

**Published:** 2024-07-05

**Authors:** Yunhao Cui, Zhihui Zhang, Yi An, Zhidan Zhong, Fang Yang, Junhua Wang, Kui He

**Affiliations:** 1 School of Mechatronics Engineering, Henan University of Science and Technology, Luoyang 471023, China; zzh@stu.haust.edu.cn (Z.Z.); zzd@haust.edu.cn (Z.Z.); yangfanghkd@haust.edu.cn (F.Y.); wangjh@haust.edu.cn (J.W.); hekui@haust.edu.cn (K.H.); 2 School of Control Science and Engineering, Dalian University of Technology, Dalian 116024, China; anyi@dlut.edu.cn; 3 Henan Intelligent Manufacturing Equipment Engineering Technology Research Center, Luoyang 471003, China; 4 Henan Engineering Laboratory of Intelligent Numerical Control Equipment, Luoyang 471003, China

**Keywords:** intelligent mining shovels, 3D point cloud semantic segmentation, memory enhancement, lightweight attention mechanism

## Abstract

The semantic segmentation of the 3D operating environment represents the key to intelligent mining shovels’ autonomous digging and loading operation. However, the complexity of the operating environment of intelligent mining shovels presents challenges, including the variety of scene targets and the uneven number of samples. This results in low accuracy of 3D semantic segmentation and reduces the autonomous operation accuracy of the intelligent mine shovels. To solve these issues, this paper proposes a 3D point cloud semantic segmentation network based on memory enhancement and lightweight attention mechanisms. This model addresses the challenges of an uneven number of sampled scene targets, insufficient extraction of key features to reduce the semantic segmentation accuracy, and an insufficient lightweight level of the model to reduce deployment capability. Firstly, we investigate the memory enhancement learning mechanism, establishing a memory module for key semantic features of the targets. Furthermore, we address the issue of forgetting non-dominant target point cloud features caused by the unbalanced number of samples and enhance the semantic segmentation accuracy. Subsequently, the channel attention mechanism is studied. An attention module based on the statistical characteristics of the channel is established. The adequacy of the expression of the key features is improved by adjusting the weights of the features. This is done in order to improve the accuracy of semantic segmentation further. Finally, the lightweight mechanism is studied by adopting the deep separable convolution instead of conventional convolution to reduce the number of model parameters. Experiments demonstrate that the proposed method can improve the accuracy of semantic segmentation in the 3D scene and reduce the model’s complexity. Semantic segmentation accuracy is improved by 7.15% on average compared with the experimental control methods, which contributes to the improvement of autonomous operation accuracy and safety of intelligent mining shovels.

## 1. Introduction

**Figure 1:**
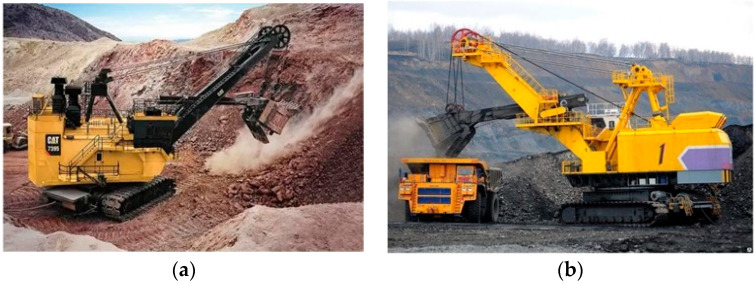
Operation of the mining shovel in the open pit mine: (**a**) mining shovels for digging operations; (**b**) loading operations with mining shovels.

Mining shovels represent the fundamental mechanical apparatus employed in the extraction of mineral resources from open pits. They integrate the two primary functions of ‘digging’ and ‘loading’, as illustrated in Figure 1 [1]. The overall performance of mining shovels significantly impacts the efficiency and economic viability of the entire mining operation, which is of paramount importance to national resource extraction and energy security [2].

Traditional mining shovels rely on manual operation, and there are many problems in the operation process. These include a low total bucket rate, high energy consumption, high failure rate, poor operational safety, and fatigue-related injury of operators. Consequently, research into the intelligence of mining shovels has become an important topic of industry development. Intelligent mining shovels can operate autonomously, utilizing advanced technologies such as artificial intelligence, robotics, automatic control, and others, to integrate functions related to sensing the operating environment, detecting the shovel’s position, planning the shovel’s chassis path, and planning the trajectory of the shovel’s digging motion.

Semantic segmentation of a 3D operation scene refers to the division of the scene into several target regions, each of which has the same or similar attributes. Each area is then assigned semantic information according to the attributes of the region and the target features. This enables the segmentation and recognition of different kinds of targets in the operation scene. The semantic segmentation of a 3D operating scene represents the foundation of the perception of an operating environment. It is also a pivotal aspect of the autonomous operation of intelligent mining shovels. The low accuracy of semantic segmentation in the 3D operation scene will render the intelligent mining shovels incapable of accurately segmenting and identifying the principal operation targets. These targets include the material pile and the mining cart. This will result in a high degree of deviation in the autonomous excavation and loading operation, and a concomitant reduction in the accuracy of autonomous operation. Concurrently, accurately segmenting and identifying objects—such as people and vehicles—within the operational environment is challenging. This can potentially lead to safety incidents. 

**Figure 2:**
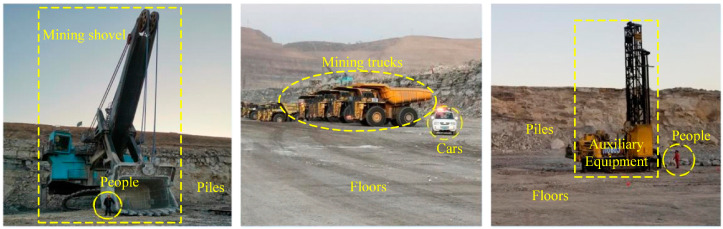
The main segmentation objects for the operating environment of the intelligent mining shovel.

The targets to be sensed in the operational environment of intelligent mining shovels include many objects including stockpiles, mining trucks, shovels, the ground, people, vehicles, auxiliary equipment, and other miscellaneous objects. These are depicted in Figure 2. Due to the extensive range of targets to be sensed and the diversity of shape structures and physical attributes, the key features of the objects are obscured by redundant features during the deep learning training process. This results in lower accuracy of semantic segmentation. Concurrently, the number of point cloud samples of different objects obtained from the actual sampling exhibits a considerable degree of unevenness, which is attributable to the influence of the characteristics of the operating conditions. It is typical for objects such as stockpiles, mining trucks, buckets, and the ground to have a more significant number of point cloud samples; whereas objects such as vehicles and people tend to have a smaller number of samples. In the case of non-dominant targets with a limited number of samples, the key features of these objects are easily overlooked during the deep learning training process, which subsequently leads to a reduction in the accuracy of semantic segmentation. Furthermore, the intricate nature of the component systems of intelligent mining shovels renders the computational task particularly onerous. The operating environment sensing system, digging trajectory planning system, and chassis path planning system all make high demands on computational resources; this necessitates the lightweight level and deployment capability of the semantic segmentation method for 3D operating scenes. Consequently, accurate and lightweight implementation of semantic segmentation of 3D operation scenes under unbalanced samples is a pivotal technical challenge. This challenge must be addressed urgently to facilitate the sensing of intelligent mining shovels within 3D operational environments.

## 2. Related Works

At present, research dedicated to semantic segmentation of intelligent mining shovels in 3D operational scenarios is relatively limited. However, certain research results have been achieved in the field of intelligent robots, unmanned vehicles, and other related fields, which have certain reference significance to this paper. This paper provides an overview of the current state of research related to semantic segmentation of 3D job scenes. It categorizes the research into two main groups: traditional machine learning methods and deep learning methods.

Based on traditional machine learning, the 3D point cloud semantic segmentation algorithm completes the segmentation and recognition of 3D operational scenes. It does this by extracting specific sample features and utilizing classifiers. The utilization of models such as Markov Random Fields [3], Random Forests [4], and Support Vector Machines [5,6] has resulted in enhanced speed and accuracy in the recognition process. In a study by Niemeyer et al. [7], a combination of Random Forest Classifier and Conditional Random Field was employed to complete the segmentation recognition of buildings in 3D operational scenes. This was achieved through the use of features such as reflection intensity and curvature. Golovinskin et al. [8] employed the graph cut method to segment the 3D operational scene. Subsequently, they extracted features—including the target location and the nearest distance to the roadside—and applied a support vector machine to classify and recognize objects such as vehicles and street lights. In their study, Yao et al. [9] extracted spectral features—as well as geometric and spatial features—from 3D point cloud data. They then employed an AdaBoost classifier to extract trees from the urban environment. Zhao et al. [10] employed a scanline algorithm to segment the 3D point cloud and extracted features, including the height and normal vector variance of the segmented surface slices. These features were then used to classify and recognize targets, such as pedestrians and trees, through the application of a support vector machine. Wang et al. [11] employed a rasterization process to remove ground points from the point cloud data, subsequently creating a local point cloud block. This block was then subjected to a reflective feature extraction process, enabling the identification and segmentation of targets such as trees and vehicles in urban environments. This was achieved through the utilization of Hough’s forest. Wang et al. [12] sampled the point cloud data at multiple scales and categorized the sampled points into different levels of point cloud clusters. They then employed a Bayesian probabilistic model and an AdaBoost classifier to recognize targets such as vehicles, pedestrians, buildings, etc., in the urban scene.

In comparison to machine learning segmentation methods, deep learning-based 3D point cloud semantic segmentation methods exhibit a reduced reliance on human-designed features, an enhanced capacity for the adaptive extraction of point cloud data features, and a higher level of semantic segmentation accuracy. The PointNet algorithm, proposed by Qi et al. [13], addresses the issues of disorder and geometric rotation invariance in point clouds. It has made significant advances in both point cloud classification and semantic segmentation. In response to the limitations of PointNet, which is unable to utilize local feature information effectively and has a poor perception of local spatial features, Qi et al. [14] proposed PointNet++. A hierarchical neural network structure is constructed, with sampling and region division occurring at each point cloud layer. The PointNet network is then employed to extract local features in each region, thereby enhancing the extraction ability of local features in the point cloud. This effectively enhances the segmentation recognition effect of point cloud targets such as walls and roofs. Jiang et al. [15] proposed PointSIFT; a 3D point cloud semantic segmentation network model based on scale-invariant feature transformations. This model encodes multiple principal directional information through directional coding units and stacks multiple coding units to obtain multi-scale features. Zhao et al. [16] proposed the PointWeb 3D point cloud semantic segmentation network model, which interconnects each point in a local neighborhood for the purpose of enhancing the extraction of local features of the point cloud. Furthermore, they proposed an adaptive feature adjustment module with the intention of enhancing the point-by-point feature representation. Wang et al. [17] applied a graph convolutional neural network to the field of 3D point cloud processing on the basis of PointNet and proposed the dynamic graph convolutional point cloud processing algorithm DGCNN. This algorithm effectively addresses the issue of the poor local spatial feature perception ability of PointNet. It simultaneously enhances the system’s point cloud classification, segmentation abilities, and robustness.

In order to provide effective convolutional operations on point clouds, and improve the local feature extraction ability of point clouds, Li et al. [18] proposed the PointCNN 3D point cloud semantic segmentation model. This model overcomes the disorder obstacle of point clouds by transforming feature input in a specific order. It converts these to order-independent features through transform matrix processing. This enables the realization of convolutional operations on point clouds. Wu et al. [19] constructed a deep convolutional semantic segmentation network, PointConv, on point clouds. This network begins with the mathematical nature of convolutional operations and regards the convolution kernel as a nonlinear function of the local coordinates of 3D points. It then learns the weight function using Multi-Layer Perception (MLP) and learns the density function using kernel density estimation. This approach effectively improves the accuracy of 3D point cloud multi-target semantic segmentation. To address the issue of sample imbalance, Francisco et al. [20] and Leng et al. [21] employed data augmentation techniques to enhance classification accuracy by augmenting the training data from small samples. Ma et al. [22] enhanced the impact of sample imbalance on the accuracy of semantic segmentation by weighting different semantic categories. In order to address the issue of insufficient feature extraction in convolutional neural networks, Hu et al. [23] proposed the SENet attention module. This module enables the adaptive correction of channel weights through the global loss function of the network; thereby improving the sufficiency of feature extraction and, in turn, the performance of the network. With regard to the issue of lightweighting of convolutional neural networks, Howard et al. [24] proposed depth-separable convolution. This approach involves splitting the conventional convolution into depth-by-depth convolution and point-by-point convolution, effectively reducing the number of network parameters and computational complexity.

Research into deep learning-based semantic segmentation methods for 3D operational scenes has progressed in application areas such as unmanned vehicles and mobile robots. This has resulted in an improvement in segmentation accuracy relative to machine learning segmentation methods. Nevertheless, the technology required for semantic segmentation in intelligent mining shovels to operate in 3D is still in its infancy. The number of point cloud samples of different kinds of objects sampled in the intelligent mining shovels operating scene exhibits a significant imbalance [25]. This imbalance is due to the influence of the operating conditions. In the case of objects with a limited number of samples, the key features of these objects are easily forgotten during the training process of deep learning, which in turn leads to a reduction in the accuracy of semantic segmentation. The existing methods are unable to address the issue of forgetting by means of data augmentation or category weighting. There is a discrepancy between the data obtained through data enhancement and the actual collected data. Furthermore, the introduction of excessive noise data during the process of data enhancement may result in a reduction in the effectiveness of model training. This is due to the fact that the category weighting will affect the training process of the network model, thereby increasing the difficulty of parameter adjustment.

Furthermore, the operational scenarios of mining shovels are characterized by a high degree of complexity, which necessitates the segmentation of a multitude of targets exhibiting a diverse range of shapes, structures, and physical attributes. During the deep learning training process, the key features of the object are more affected by redundant features, which affects the adequacy of the key feature extraction and results in lower semantic segmentation accuracy. In the meantime, the intricate composition system of intelligent mining shovels requires a lightweight configuration for the 3D point cloud semantic segmentation network model. This is due to the substantial computational load and the need for deployment capability. Furthermore, the existing methods are unable to accurately and efficiently perform semantic segmentation of the three-dimensional operation scene of intelligent mining shovels. This is especially true under unbalanced samples.

In order to address the aforementioned issues, this paper presents a 3D point cloud semantic segmentation network model that employs memory enhancement and a lightweight attention mechanism. The model is based on the memory enhancement mechanism, which effectively alleviates the feature-forgetting problem of non-dominant targets caused by the imbalance in the number of point cloud samples of different kinds of targets. This is achieved through the channel attention mechanism, which improves the adequacy of key feature extraction in the point cloud. Finally, the lightweight mechanism reduces the number of model parameters. The network model proposed in this paper can accurately and efficiently segment the 3D operation scene of intelligent mining shovels, even when the samples are imbalanced.

## 3. Semantic Segmentation of 3D Operational Scenes Based on Memory Enhancement and Lightweight Attention Mechanisms

This paper presents a semantic segmentation network model for intelligent mining shovels with 3D operation scenes constructed using PointConv as the basic framework. The main reason for using PoincConv is that it can efficiently perform convolution operations on non-uniformly sampled 3D point cloud data, with both translation invariance and point sequential permutation invariance. The overall network structure, along with the memory module, channel attention module, and network lightweight mechanism are described in this section.

### 3.1. Overall Network Structure

**Figure 3:**
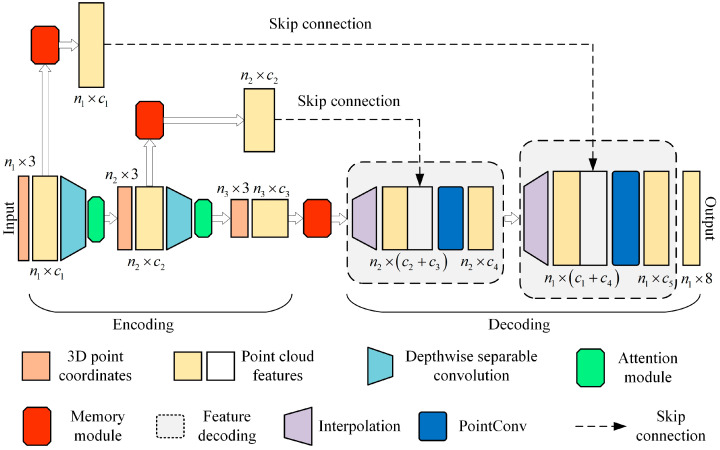
Overall architecture of MALMConv.

In order to address the challenges of sample imbalance, insufficient key feature extraction, and model deployment encountered in the semantic segmentation of smart mining shovels, this paper presents a network model for semantic segmentation based on the memory module and the lightweight attention mechanism, MALMConv (PointConv + Memory Module + Attention Module + Lightweight Module, MALMConv). This model offers an accurate and lightweight implementation of intelligent mining shovels’ semantic segmentation under unbalanced samples, as illustrated in Figure 3.

The MALMConv employs PointConv as its fundamental framework, adding memory modules between the encoder and decoder of the original PointConv framework. Furthermore, corresponding memory modules are incorporated into the hop-connected branched network. Consequently, the enhancement of all the target point clouds’ key semantic features extracted by the encoder is achieved prior to their entry into the decoder. The introduction of the memory module has resulted in a significant improvement in the accuracy of semantic segmentation. This improvement is particularly notable in intelligent mining shovels operating in 3D environments. This is due to the fundamental mitigation of forgetting the training of non-dominant target features caused by the unbalanced number of target point cloud samples. Furthermore, in order to enhance the efficacy of PointConv’s key feature extraction in the encoding stage, a channel attention module has been incorporated to dynamically adjust the weights of distinct feature channels, thereby optimizing the expression of pivotal key point cloud features pertinent to the current task. This, in turn, serves to reinforce the precision of the semantic segmentation of the 3D point clouds. Concurrently, in order to optimize the utilization of limited computational resources and enhance the deployment capabilities of the model, depth separable convolution is employed in lieu of conventional convolution during the coding stage. This approach results in a reduction in the number of model parameters and an improvement in the model’s lightweight nature, as well as its actual engineering deployment capability. Typically, depth-separable convolution can be employed in conjunction with the attention module to extract point cloud features. This involves applying depth-separable convolution initially, followed by adjusting the weights of feature channels based on the channel attention module.

### 3.2. Memory Module

**Figure 4:**
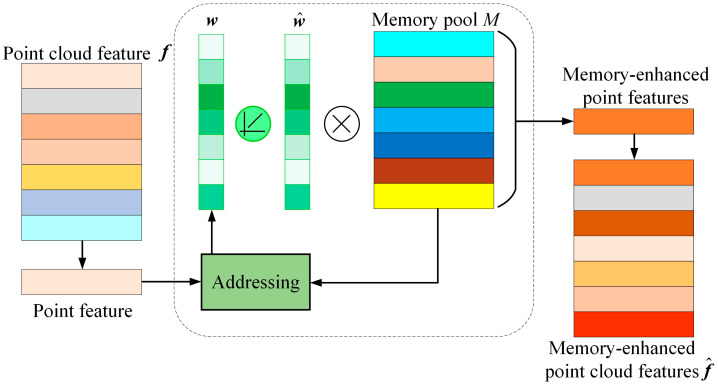
Memory module. (The different colors are to distinguish the feature vectors).

As illustrated in Figure 4, the memory module is composed of two primary components: a memory pool and an an addresser. The memory pool serves to record all the key semantic features of the target to be segmented, while the addresser is employed to access the most relevant key semantic features stored in the memory pool. The memory pool comprises a number of memory slots, each of which is designed to record the stored features of a target to be segmented. Upon receipt of an input feature, the addresser will calculate the degree of similarity between the input feature and each stored feature within the memory pool. This value will then be used as a weighted average of all stored features within the memory pool, with the aim of ultimately obtaining the memory feature corresponding to the input feature. The ReLU activation function employed in the calculation of weights serves to suppress smaller weights, thereby enabling the retention of only those semantic features exhibiting greater similarity to the input features. This process is essential for the successful implementation of semantic segmentation in relation to the category labels. 

During training, the memory content in the memory pool is dynamically updated along with the encoders and decoders throughout the segmentation network, driven by the goal of the 3D point cloud semantic segmentation task. Due to the sparse addressing strategy, the memory module is motivated to efficiently use the memory slots in the memory pool to store the key semantic features of the target point cloud acquired during the batch sample learning process. During training, the memory module dynamically learns and remembers all the key semantic features of the targets to be segmented. In the test phase, the learned memories are fixed. Based on the input features, the memory module retrieves the most relevant key semantic features stored in the memory pool and feeds the memory-enhanced point cloud semantic features to the decoder for 3D point cloud semantic segmentation. Therefore, under the action of the memory module, the point cloud semantic features of the non-dominant targets can be enhanced by retrieving the key semantic features stored in the memory pool, thus alleviating the problem of forgetting the features of the non-dominant targets and improving the accuracy of semantic segmentation of intelligent mining shovels in 3D operational scenes.

The memory pool is designed as a matrix $M\in R^{N\times D}$, where *N* is the number of memory slots and *D* is the dimension of the memory feature. Typically, *N* is the number of the semantic categories, and D is the dimensional size of the input point cloud features. ***f***. $m_{i}\in R^{1\times D}$ is the *i*th row vector of the memory pool *M*, representing the memory contents in the *i*th memory slot, each corresponding to a semantic category. The memory pool can be viewed as a dictionary that continuously records and updates the key semantic features of the target point cloud to be segmented during the training process.

The addresser is mainly used to obtain the addressing vector and retrieve the key semantic feature $\hat{f}$ most relevant to the input feature ***f*** from the memory pool based on this addressing vector to achieve memory enhancement of the input feature. First, the addresser obtains the address weight *w_i_* by calculating the similarity between the input feature ***f*** and the key semantic feature ***m****_i_* stored in the memory pool, where *w_i_* is the *i*th element of the address vector ***w***. The address weight *w_i_* is calculated as follows:

(1) $$w_{i}=\frac{\exp\left(\tau \left(f,m_{i}\right)\right)}{\sum_{i=1}^{N}\exp\left(\tau \left(f,m_{i}\right)\right)},$$

where $\tau \left(f,m_{i}\right)$ is a metric function used to compute the feature similarity between the input point cloud features ***f*** and the key semantic features mi stored in the memory pool. $\tau \left(f,m_{i}\right)$ is computed as follows:

(2) $$\tau \left(f,m_{i}\right)=\frac{\sum_{j=1}^{D}\left(f_{j}-\bar{f}\right)\left(m_{ij}-{\bar{m}}_{i}\right)}{\sqrt{\sum_{j=1}^{D}{\left(f_{j}-\bar{f}\right)}^{2}}\sqrt{\sum_{j=1}^{D}{\left(m_{ij}-{\bar{m}}_{i}\right)}^{2}}},$$

where the means $\bar{f}$ and ${\bar{m}}_{i}$ are calculated as follows:

(3) $$\bar{f}=\frac{1}{D}\sum_{j=1}^{D}f_{j}$$

(4) $${\bar{m}}_{i}=\frac{1}{D}\sum_{j=1}^{D}m_{ij}$$

To further suppress the influence of irrelevant memory content on the semantic features of the memory-enhanced point cloud, and to make the memory module more efficient at storing the key features, the addressing weight *w_i_* is sparsified using the ReLU function. The sparsified address weights ${\tilde{w}}_{i}$ are computed as follows:

(5) $${\tilde{w}}_{i}=\frac{\max\left(w_{i}-\beta ,0\right)\cdot w_{i}}{\left|w_{i}-\beta \right|+\varepsilon }$$

where max(⋅,0) is the ReLU function, β is the shrinkage threshold, and ε is the moderating factor. After the sparsification process, the sparsified addressing weight ${\tilde{w}}_{i}$ is normalized. The normalized weight ${\hat{w}}_{i}$ is calculated as follows:

(6) $${\hat{w}}_{i}=\frac{{\tilde{w}}_{i}}{\sum_{i=1}^{N}\left|{\tilde{w}}_{i}\right|}$$

Finally, the key semantic features $\hat{f}$ of the point cloud retrieved from the memory module are computed as follows:

(7) $$\hat{f}=\sum_{i=1}^{N}{\hat{w}}_{i}m_{i},$$

By constructing the memory module, the storage of the key semantic features of the point cloud of the target to be segmented is achieved, which fundamentally alleviates the feature forgetting problem of the non-dominant target during the training process of the semantic segmentation network, and effectively improves the semantic segmentation accuracy rate of intelligent mining shovels in the 3D operation scene.

### 3.3. Channel-Wise Attention Module

**Figure 5:**
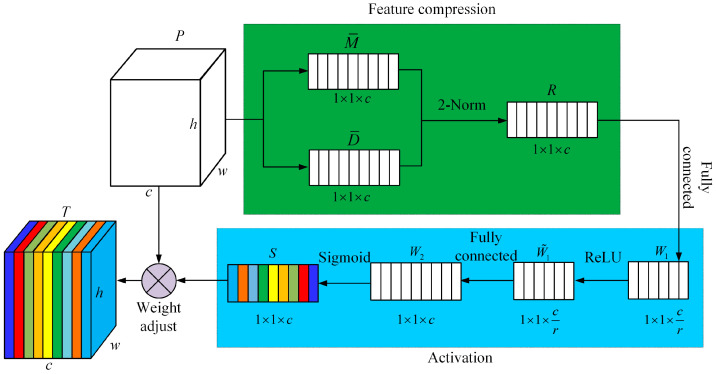
Channel-wise attention module. (Different colors indicate different feature vectors after weight adjustment).

The framework of the feature channel attention module is shown in Figure 5. It consists of three key components: feature compression, excitation, and weight adjustment. 

#### 3.3.1. Feature Compression

On each feature channel, the input point feature $P=\left\{P_{k}|1\leq k\leq c\right\}$ is compressed into a real number by feature compression, and the real number is used as the channel descriptor, where *k* denotes the channel sequence number. $\bar{M}=\left\{\mu_{k}|1\leq k\leq c\right\}$ denotes the channel descriptor based on the mean value over all feature channels, where the channel descriptor $\mu_{k}$ corresponding to feature channel *k* is calculated as follows:

(8) $$\mu_{k}=\frac{1}{h\times w}\sum_{i=1}^{h}\sum_{j=1}^{w}U_{k}\left(i,j\right),$$

where h×w is the spatial dimension of the input features. $\bar{D}=\left\{\sigma_{k}|1\leq k\leq c\right\}$ is the standard deviation-based channel descriptor over all feature channels, where the channel descriptor $\sigma_{k}$ corresponding to feature channel *k* is calculated as follows:

(9) $$\sigma_{k}=\sqrt{\frac{1}{h\times w}\sum_{i=1}^{h}\sum_{j=1}^{w}{\left(U_{k}\left(i,j\right)-\mu_{k}\right)}^{2}},$$

$R=\left\{r_{k}|1\leq k\leq c\right\}$ denotes the two-paradigm number based on two statistically significant features—mean and standard deviation—over all feature channels, where the channel descriptor $r_{k}$ corresponding to feature channel *k* is computed as follows:

(10) $$r_{k}=\sqrt{\mu_{k}^{2}+\sigma_{k}^{2}},$$

The feature compression operation compresses and aggregates multiple spatial dimensional features on each feature channel to generate a channel descriptor. The channel descriptor has a global information-sensing field that can effectively reflect the average value and range of variation of all features on the feature channel.

#### 3.3.2. Activation

The channel descriptor *R* is obtained by the first fully connected layer operation to obtain the weight coefficient $W_{1}\in R^{1\times 1\times c/r}$, which is activated using the ReLU function to obtain the weight coefficient ${\tilde{W}}_{1}\in R^{1\times 1\times c/r}$, where *r* denotes the scaling factor of the gating mechanism. Adopting ReLU as the activation function helps to alleviate the vanishing gradient problem and improve the convergence speed. Then, the weight coefficients $W_{2}\in R^{1\times 1\times c}$ are obtained by the second fully connected layer operation, and the weight coefficients $S\in R^{1\times 1\times c}$ are obtained after activation using the sigmoid function. The sigmoid is used as the activation function, which is mainly used to obtain normalized weight coefficients with values ranging from 0 to 1. The weight coefficient $S\in R^{1\times 1\times c}$ is used to measure the importance of each feature channel for the semantic segmentation task and is calculated as follows:

(11) $$S=\beta \left(W_{2}\delta \left(W_{1}R\right)\right),$$

where δ is the ReLU activation function and β is the Sigmoid activation function.

#### 3.3.3. Weighting

The input features are weighted using the weight coefficients $S\in R^{1\times 1\times c}$. After fitting, the feature $T=\left\{T_{k}|1\leq k\leq c\right\}$ is obtained, and the weight calibration is calculated as follows:

(12) $$T_{k}=s_{k}P_{k},$$

By constructing the channel attention module to adjust the weights of the features, the weights can be calibrated according to the magnitude of the role of different feature channels. This can stimulate the useful information that is more critical to the current task, and suppress the irrelevant information. Then, it can improve the exploitation of the key features within the limited computational resources, and contribute to the improvement of the semantic segmentation accuracy.

### 3.4. Depthwise Separable Convolution

**Figure 6:**
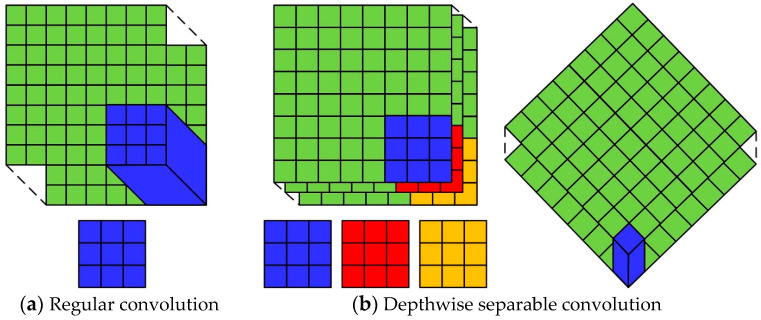
Regular convolution and depthwise separable convolution.

In the 3D point cloud semantic segmentation network proposed in this paper, depthwise separable convolution is employed instead of regular convolution. The objective is to reduce the number of parameters of the model. This enhances the lightweight nature of the model, thereby facilitating its deployment in practical applications. As illustrated in Figure 6a, conventional convolution employs a convolution kernel with an identical number of channels to those of the input data, thus enabling the execution of both cross-channel operations and spatial operations. Figure 6b illustrates that depthwise separable convolution decomposes the regular convolution operation into depth-by-depth convolution and point-by-point convolution. Firstly, depth-by-depth convolution performs separate independent convolution operations on each feature channel. Secondly, point-by-point convolution employs the 1×1 convolution operation to combine the depth-by-depth convolution outputs on different channels in order to achieve cross-channel operations. The decomposition of cross-channel and spatial operations enables the reduction of the number of parameters in semantic segmentation network models. This is achieved through the use of depthwise separable convolution.

## 4. Experimental Results and Analysis

This section presents the establishment of a semantic segmentation dataset of intelligent mining shovels for 3D operation scenes. Subsequently, a comparison is conducted between MALMConv and other representative semantic segmentation networks. This demonstrates the segmentation effect of the semantic segmentation network proposed in this paper. Finally, a series of ablation experiments are conducted to validate the memory module, the channel attention module, and the lightening mechanism, among other key technologies.

### 4.1. Dataset Creation

Relying on the State Key Laboratory of Mining Equipment and Intelligent Manufacturing, this thesis discusses the demand for semantic segmentation of intelligent mining shovels for 3D operation scenes, and reasonably arranges the experimental scene with reference to the real operating environment of mining shovels in the mine site. By modifying the spatial arrangement of objects—such as shovels, people, mining trucks, boxes, and so forth—as well as the composition and topography of the pile of materials and ground surface, and by varying the sampling position and angle, it is possible to expand the range of experimental scenarios and enhance the generalizability of the dataset. Concurrently, to enhance the dataset’s practicality, the frequency of different targets in the laboratory scenario is reasonably adjusted through field research of the real operating conditions in the mine site. This is done to ensure that the dataset can effectively reflect the imbalance of the number of point cloud samples. It highlights the differences in sample numbers for different types of targets in the mine operating scenario.

**Figure 7:**
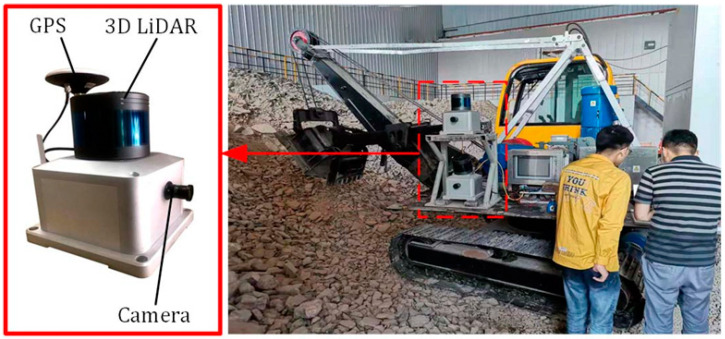
The 3D operating environment sensing system.

We conducted point cloud data acquisition at the State Key Laboratory of Mining Equipment and Intelligent Manufacturing in 2022. The equipment used for data collection is our self-developed 3D operating environment sensing system, which is shown in Figure 7. 

**Figure 8:**
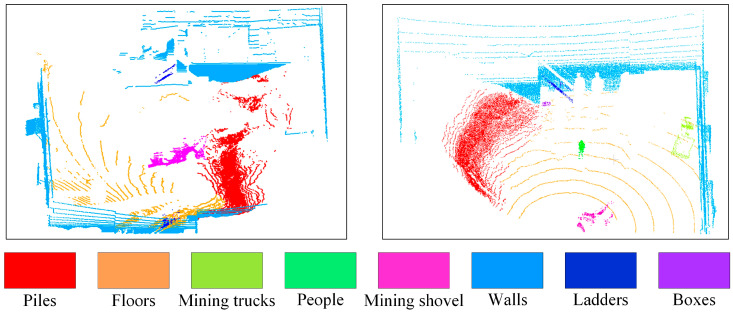
3D point cloud semantic segmentation data set for the intelligent mining shovels.

These data are then used to establish a semantic segmentation dataset of the 3D operating scene of intelligent mining shovels through manual annotation. Figure 8 illustrates the typical 3D point cloud data following manual annotation. The dataset comprises semantic targets to be segmented, including eight types of target objects. These include 1:7 scaled intelligent mining shovels prototypes, mining trucks, piles, floors, people, walls, ladders, boxes, and other items. The dataset contains a wealth of operational objects and targets to be segmented. Among the aforementioned targets, five types are of particular importance: mining shovels, mining trucks, stockpiles, ground, and people. These are the most crucial semantic targets to be segmented in the real operating scenarios of mining shovels at the mine site. Consequently, the model training and experimental validation based on this dataset are of great significance. They are crucial for subsequent research on the perception method of intelligent mining shovels in the mine site. Furthermore, in order to ensure the robustness of the algorithm, three types of laboratory scenario targets—such as walls, ladders, and boxes—are also semantically labeled in the dataset as targets to be segmented, in addition to considering the existence of some clutter in the real operating scenarios of mines.

We collected 2200 point clouds, of which 2000 point clouds are used as the training set and 200 point clouds are used as the test set. The number of point clouds corresponding to different targets in the practice set is shown in Table 1, in which the number of samples corresponding to targets such as piles, floors, walls, ladders, electric shovels, and mining trucks is relatively high and is the dominant target. The number of samples corresponding to people and boxes is relatively small and are non-dominant targets. The dataset covers the main targets to be sensed in the actual operating scenarios of intelligent mining shovels in the mining site; and the physical attributes are diverse, and the sample imbalance problem is significant, so it can effectively respond to the problems existing in the actual operating scenarios of intelligent mining shovels. The study of semantic segmentation algorithms for 3D operational scenes of intelligent mining shovels based on this dataset is of great significance. It is crucial for the next stage of conducting experimental research in the mining field.

### 4.2. Experimental Setup and Performance Evaluation Index

The programming language used for the experiments in this paper is Python 3.6, the deep learning framework is TensorFlow 1.12, and the GPU is Tesla P100. The training optimizer used for MALMConv is ADAM; with an initial learning rate of 0.001, a momentum of 0.9, a batch size of 8, a decay step of 200,000, and a decay rate of 0.7. 

The performance evaluation metrics used in the semantic segmentation experiments in this paper include:

(1) Single class accuracy (SAC): Denotes the semantic segmentation accuracy for each semantic category. For any semantic category c, SAC is calculated as follows:
  (13) $$\mathrm{SAC}=\frac{1}{n_{c}}\sum_{i=1}^{n_{c}}\frac{N_{ic}^{t}}{N_{ic}},$$

The notation $n_{c}$ represents the number of point clouds in the dataset that belong to semantic category c. The symbol $N_{ic}$ denotes the ideal value of the number of points corresponding to semantic category c in the *i*th sub-point cloud. The symbol $N_{ic}^{t}$ represents the number of points in the *i*th sub-point cloud correctly predicted for semantic category c.

(2) Average accuracy (AAC): Denotes the mean of semantic segmentation accuracy for all semantic categories. For the eight semantic categories in the dataset of this paper, the AAC is calculated as follows:

(14) $$\mathrm{AAC}=\sum_{j=1}^{8}\frac{{\mathrm{SAC}}_{j}}{8},$$

where $SAC_{j}$ is the semantic segmentation accuracy for the *j*th semantic category.

(3) Number of parameters (Np): this indicates the number of weight parameters in the semantic segmentation network model. It is an important evaluation index to measure the level of model lightness and affect the difficulty of model deployment.

### 4.3. Semantic Segmentation Accuracy Comparison Experiments and Visualizations

In order to verify the performance of MALMConv, the 3D point cloud semantic segmentation network proposed in this paper, PointNet, PointNet++, DGCNN and PointConv are used as comparison methods. The comparison experiments are conducted to validate the semantic segmentation of intelligent mining shovels. These experiments are based on the semantic segmentation dataset of the 3D operation scene established in this paper. Table 2 shows the single-category semantic segmentation accuracy for eight categories of targets such as piles, floors, mining trucks, people, mining shovels, walls, ladders, and boxes. 

As illustrated in Table 2, the proposed MALMConv exhibits the most optimal overall performance; achieving the highest semantic segmentation accuracy on almost all objectives. The semantic segmentation accuracy of MALMConv for two non-dominant targets—namely, the human and the box—exhibits a marked improvement compared to other methods. The primary rationale for this approach is that the memory module can effectively address the issue of non-dominant target features being forgotten during the training process, thereby enhancing the accuracy of semantic segmentation of 3D point clouds. Concurrently, the attention module—which is based on the statistical characteristics of the feature channels—can adaptively learn the weights of different feature channels. This enables the limited computational resources to be used to filter the feature information that is more critical to the current task. It also improves the adequacy of the feature expression. This, in turn, further improves the accuracy rate of semantic segmentation of 3D point clouds.

PointNet exhibits the lowest level of semantic segmentation accuracy among the four methods under comparison. This is primarily attributable to PointNet not considering the interrelationships between neighboring points. Additionally, it does not extract and utilize the local neighborhood features of the point cloud. This ultimately affects the accuracy of the semantic segmentation of the point cloud in a complex scene. The semantic segmentation accuracy of PointConv is higher than that of PointNet, PointNet++, and DGCNN. This is primarily due to the effective implementation of convolutional operations on 3D point clouds by PointConv, which enables the extraction and exploitation of local neighborhood features of the point cloud, thus improving the semantic segmentation accuracy of the point cloud. However, compared with the MALMConv proposed in this paper, PointConv is more susceptible to the imbalanced number of samples. Furthermore, it is relatively straightforward to overlook the point cloud features of the non-dominant targets during the training process, which subsequently reduces the semantic segmentation accuracy rate of the non-dominant targets. Additionally, PointConv is unable to model the relationship between the different feature channels explicitly, and it is also unable to differentiate the importance of the different feature channels, which ultimately leads to a reduction in the semantic segmentation accuracy.

**Figure 9:**
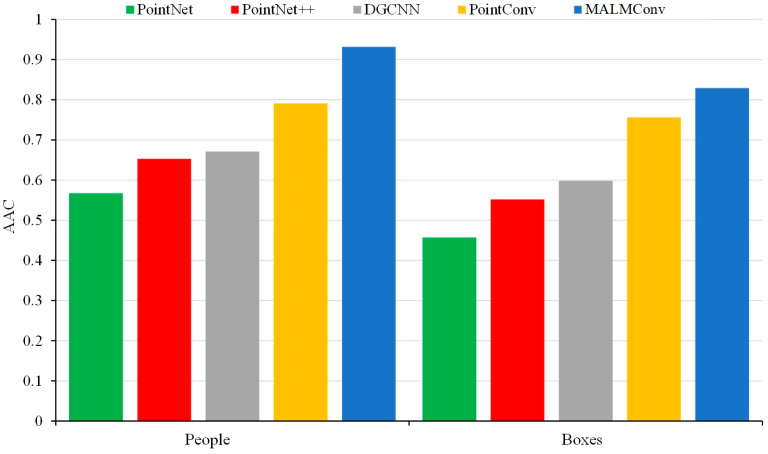
Comparison of semantic segmentation accuracy of non-dominant objects.

Figure 9 compares the semantic segmentation accuracies of two non-dominant targets based on the five segmentation methods. It can be observed that the MALMConv network proposed in this paper is efficacious in improving the accuracy of semantic segmentation of non-dominant targets. This is in comparison to PointNet, PointNet++, DGCNN, and PointConv. For the non-dominant target of people, MALMConv improved semantic segmentation accuracy by 36.5% compared to PointNet, 27.9% compared to PointNet++, 26.1% compared to DGCNN, and 14.1% compared to PointConv. For the non-dominant goal of boxes, MALMConv improved semantic segmentation accuracy by 37.2% compared to PointNet, 27.7% compared to PointNet++, 23.1% compared to DGCNN, and 7.3% compared to PointConv. In conclusion, it can be demonstrated that MALMConv is an effective method for alleviating the forgetting problem of non-dominant target point cloud features during the training process. This results in an improvement in the accuracy of 3D point cloud semantic segmentation.

**Figure 10:**
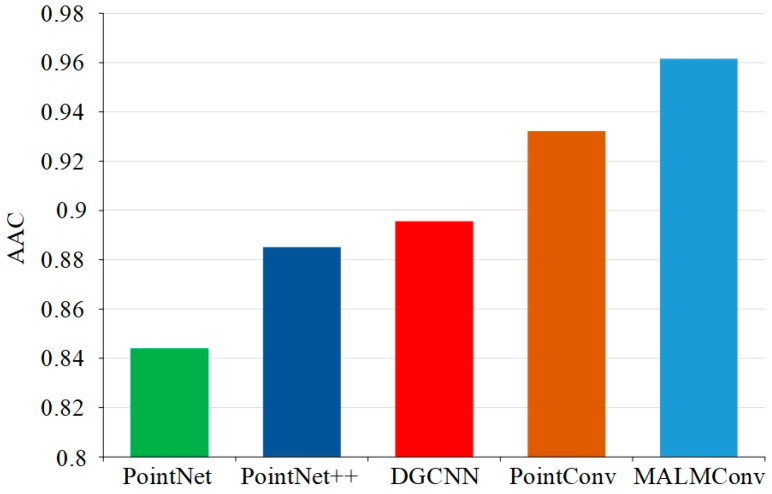
Comparison of average accuracy for semantic segmentation.

Figure 10 presents a comparison of the average accuracy of semantic segmentation across the five segmentation methods on all the point cloud samples in the test set. It can be observed that the MALMConv proposed in this paper exhibits a notable improvement in terms of average accuracy of semantic segmentation. This is in comparison to PointNet, PointNet++, DGCNN, and PointConv. Among the evaluated methods, MALMConv demonstrated superior performance in semantic segmentation, with an average accuracy improvement of 11.6% compared to PointNet, 7.6% compared to PointNet++, 6.6% compared to DGCNN, and 2.8% compared to PointConv. In summary, it can be proved that MALMConv can achieve high semantic segmentation accuracy for different sample numbers and different kinds of 3D point cloud targets. This can effectively improve intelligent mining shovels’ perception and recognition ability for complex operation scenes.

**Figure 11:**
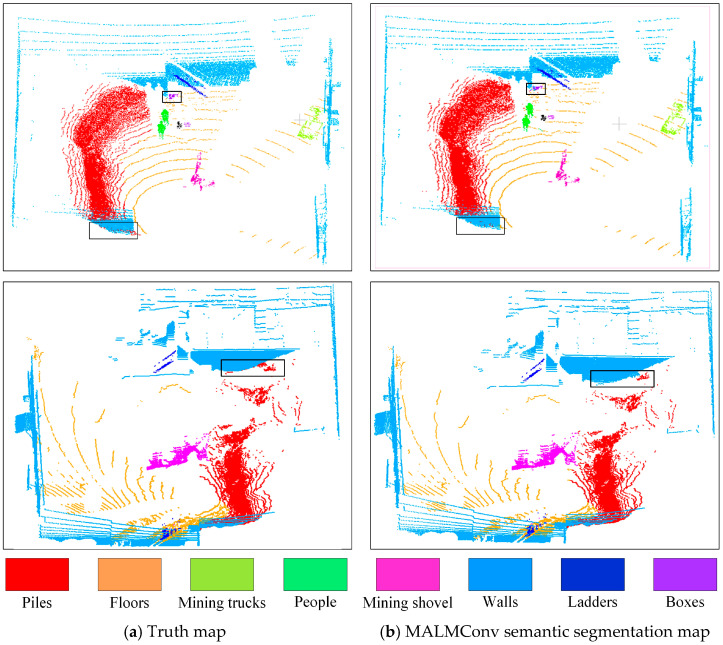
Visual display of the semantic segmentation results with MALMConv.

To more intuitively demonstrate the semantic segmentation effect of the MALMConv proposed in this paper, two typical 3D point cloud data of intelligent mining shovels operation scenes are selected. These are from the semantic segmentation test set established based on the State Key Laboratory of Mining Equipment and Intelligent Manufacturing for the visual demonstration. These data are shown in Figure 10. Among them, Figure 11a shows the true value map, and Figure 11b shows the semantic segmentation map based on MALMConv. The figure shows that MALMConv can accurately segment the semantic targets in the 3D operation scene of intelligent mining shovels, and the 3D point cloud semantic segmentation effect is very close to the actual value graph. Where the black box shows the segmentation inaccuracies.

### 4.4. Ablation Study

This section presents ablation studies conducted to validate the roles of memory, channel attention, and lightweight modules in the semantic segmentation task of intelligent mining shovels for 3D operational scenes. To achieve this objective, PointConv is employed as the fundamental network framework, adding a memory module for enhancement, and resulting in the generation of MMConv (PointConv + Memory Module, MMConv). Conversely, incorporating a memory module and attention module leads to MAMConv (PointConv + Memory Module + Attention Module, MAMConv). In contrast, the combination of a memory module, attention module, and lightweight module gives rise to MALMConv.

Table 3 demonstrates the single-category semantic segmentation accuracies and average accuracies of PointConv, MMConv, MAMConv, and MALMConv on eight categories of objects such as piles, floors, mining trucks, people, mining shovels, walls, ladders, and boxes. Table 4 shows the number of parameters for PointConv, MMConv, MAMConv, and MALMConv.

Table 3 shows that, after introducing the memory module alone, the semantic segmentation accuracy of MMConv is improved to varying degrees in all target categories relative to PointConv. Especially for non-dominant targets, MMConv’s improvement in semantic segmentation accuracy is significant. For the non-dominant target of people, the semantic segmentation accuracy of MMConv is improved by 15.7% compared to PointConv; for the non-dominant target of boxes, the semantic segmentation accuracy of MMConv is improved by 7.2% compared to PointConv. It can be proved through experiments that the memory module can effectively alleviate the forgetting problem of non-dominant target point cloud features in the training process, and improve the accuracy of 3D point cloud semantic segmentation of non-dominant targets in intelligent mining shovels operation scenarios.

With the introduction of both the memory module and the channel attention module, the semantic segmentation accuracy of MAMConv is further improved to varying degrees in terms of overall object categories relative to MMConv. Among them, the most obvious enhancement effect is the target object of boxes, where the semantic segmentation accuracy of MAMConv is improved by 12.7% compared with PointConv, and the semantic segmentation accuracy of MAMConv is improved by 5.5% compared with MMConv. It can be demonstrated through experiments that the channel attention module can learn the weights of different feature channels adaptively. It improves the adequacy of feature expression, further enhancing the accuracy of 3D point cloud semantic segmentation for each sample target in the intelligent mining shovels operation scene.

A comprehensive look at Table 3 and Table 4 shows that with the simultaneous introduction of the memory module, the channel attention module, and the lightweight mechanism, the comprehensive performance of MALMConv achieves an optimal balance in terms of the semantic segmentation accuracy, the number of model parameters, and the running time. MALMConv gets a significant reduction in both the number of parameters and the running time compared with other networks. The experiment proves that using a lightweight mechanism can effectively improve the lightweight level of the model and its ability to be deployed in practical applications. Regarding semantic segmentation accuracy, MALMConv is significantly improved compared to PointConv and similar to MMConv.

The three key technologies—the memory module, the channel attention module, and the lightweight mechanism—can be used individually or in a free combination. In the practical application process, appropriate choices can be made according to the needs of actual working conditions. In this case, the highest semantic segmentation accuracy of the model is achieved when both memory and channel attention modules are used. Still, the number of model parameters and computations increased. This combination of modules can be preferred when the computational resources of the on-board industrial controller allow it. This can maximize the accuracy of 3D point cloud semantic segmentation. With the simultaneous use of a memory module, a channel attention module, and a lightweight mechanism, the model achieves an optimal balance between semantic segmentation accuracy and lightweight. In the limited computational resources of on-board industrial controllers, this combination of modules can be preferred to improve the underlying network model to achieve a higher semantic segmentation accuracy under limited computational resources. In addition, these key techniques have a wide range of applicability. They can be used to improve a wide range of representative 3D point cloud semantic segmentation network frameworks such as PointNet, PointNet++, DGCNN, PointConv, and so on.

## 5. Conclusions

In this paper, we study the semantic segmentation method of 3D operation scenes of intelligent mining shovels. This is based on memory enhancement and a lightweight attention mechanism. The main contributions are summarized as follows:

(1) Aiming at the problem of low semantic segmentation accuracy of small sample targets, we propose a memory enhancement learning mechanism to fundamentally alleviate the problem of forgetting the point cloud features of non-dominant targets during training;
(2) Aiming at the problem of low accuracy of semantic segmentation due to the interference of key features by redundant features, we construct an attention module based on channel statistical feature analysis, which adaptively adjusts feature weights during training to improve the adequacy of key feature expression;
(3) To improve the model lightweight level and engineering deployment ability, we use depth-separable convolution instead of traditional convolution, reducing the number of parameters of 3D point cloud semantic segmentation network model;
(4) A semantic segmentation network for intelligent mining shovels in 3D operation scenes has been constructed based on the memory module, channel attention module, and lightweight mechanism. The model has been trained and tested, and the results demonstrate that it can effectively improve the semantic segmentation accuracy and engineering deployment capability of intelligent mining shovels in 3D operation scenes. The average improvement in semantic segmentation accuracy is 7.15%, contributing to enhanced autonomous operations and the safety of intelligent mining shovels.

## Figures and Tables

**Table 1:** Statistics of point cloud samples for different semantic segmentation objects.

Semantic Category	Piles	Floors	Mining Trucks	People	Mining Shovel	Walls	Ladders	Boxes
Training Set	1999	2000	1185	200	1296	2000	1987	100
Testing Set	200	200	100	20	100	200	200	20

**Table 2:** Semantic segmentation accuracy based on different segmentation methods.

Method	Piles	Floors	Mining Trucks	People	Mining Shovel	Walls	Ladders	Boxes
PointNet	0.982	0.957	0.937	0.567	1.000	0.989	0.873	0.457
PointNet++	0.987	0.965	0.973	0.653	1.000	0.990	0.957	0.552
DGCNN	0.992	0.940	0.980	0.671	1.000	0.997	0.978	0.598
PointConv	0.988	0.966	0.974	0.791	1.000	0.998	0.989	0.756
MALMConv	0.990	0.969	0.980	0.932	1.000	0.998	0.990	0.829

**Table 3:** Ablation studies 1.

Method	Average Accuracy	Piles	Floors	Mining Trucks	People	Mining Shovel	Walls	Ladders	Boxes
PointConv	0.933	0.988	0.966	0.974	0.791	1.000	0.998	0.989	0.756
MMConv	0.963	0.990	0.969	0.978	0.948	1.000	0.998	0.990	0.828
MAMConv	0.972	0.993	0.970	0.987	0.950	1.000	0.998	0.991	0.883
MALMConv	0.961	0.990	0.969	0.980	0.932	1.000	0.998	0.990	0.829

**Table 4:** Ablation studies 2.

Method	Number of Parameters (M)	Time (s)
PointConv	21.06	0.260
MMConv	21.67	0.263
MAMConv	21.76	0.264
MALMConv	16.18	0.248

## Data Availability

The original contributions presented in the study are included in the article, further inquiries can be directed to the corresponding author.
